# BNT162b2 Booster Vaccination Induced Immunity against SARS-CoV-2 Variants among Hemodialysis Patients

**DOI:** 10.3390/vaccines10060967

**Published:** 2022-06-17

**Authors:** Michal Herman-Edelstein, Naomi Ben-Dor, Timna Agur, Tali Guetta, Annat Raiter, Eshcar Meisel, Weaam Alkeesh, Yaacov Ori, Benaya Rozen-Zvi, Boris Zingerman

**Affiliations:** 1 The Felsenstein Medical Research Center, Nephrology Lab, Petah Tikva 4941492, Israel; naomib3@clalit.org.il (N.B.-D.); taliguetta@mail.tau.ac.il (T.G.); weaamal@clalit.org.il (W.A.); bnaiar@clalit.org.il (B.R.-Z.); 2 Rabin Medical Center, Department of Nephrology, Petah Tikva 4941492, Israel; timna.agur@clalit.org.il (T.A.); eshcarme@clalit.org.il (E.M.); yakovo@clalit.org.il (Y.O.); borisz@clalit.org.il (B.Z.); 3 Sackler Faculty of Medicine, Tel Aviv University, Tel Aviv 6997801, Israel; araiter@tauex.tau.ac.il; 4 Felsenstein Medical Research Center, Breast Cancer Research Lab, Petah Tikva 4941492, Israel

**Keywords:** COVID-19 vaccine, hemodialysis patients, COVID-19 variants: B.1.617.2 (Delta) and B.1.1.529 (Omicron)

## Abstract

**Background:** The emergence of new SARS-CoV-2 variants, which evade immunity, has raised the urgent need for multiple vaccine booster doses for vulnerable populations. In this study, we aimed to estimate the BNT162b2 booster effectiveness against the spread of coronavirus variants in a hemodialysis population. **Methods:** We compared humoral and cell-mediated immunity in 100 dialysis patients and 66 age-matched volunteers, before and 2–3 weeks following the first booster vaccine dose. Participants were assessed for anti-spike (RBD) antibody titer, neutralizing antibodies against B.1.617.2 (Delta) and B.1.1.529 (Omicron) variants, spike-specific T-cell responses by FACS and infection outbreak after the first and second booster. **Results:** Anti-spike antibody titer was significantly increased following the booster, with reduced humoral and cellular response in the dialysis patients. Neutralizing antibody levels increased significantly after the booster dose, with an inferior effect (≤2 fold) against Omicron compared with the Delta variant. Furthermore, CD4+ and CD8+ T-cell activation by Delta spike protein was preserved in 70% of PBMCs from the dialysis patients. A second booster dose tended to reduce breakthrough infections in the dialysis patients. **Conclusions:** Until the release of an updated vaccine, BNT162b2 booster doses will improve the humoral and cell-mediated immunity against variants. These findings support the importance of repetitive booster doses for hemodialysis patients.

## 1. Introduction

Hemodialysis patients appear to be a highly vulnerable population to severe acute respiratory syndrome coronavirus 2 (SARS-CoV-2) infections, with high morbidity and a 20% mortality rate [1,2].

As we and others have reported, the effectiveness of BNT162b2 (Pfizer–BioNTech) is reduced in this population. Only 79.8% of hemodialysis patients maintained seropositivity levels six months from the vaccination compared with 98.8% in the control group [3,4,5]. Comorbid medical conditions, mainly malnutrition and diabetes, are common in the dialysis population and are likely to contribute to the low response rate to the vaccines. Furthermore, both hemodialysis patients and kidney transplant recipients show rapid waning of mRNA vaccine protection against SARS-CoV-2 infection with a rapid decline in vaccine-induced antibodies [3,4,6,7]. In accordance with this, several studies that assessed vaccine-induced neutralizing antibodies against SARS-CoV-2 have shown late induction and a decreased level of the neutralizing antibody titer in dialysis patients [8,9].

As with other RNA viruses, SARS-CoV-2 is subject to a high rate of mutational changes. During a pandemic, the concern is that a high viral spread with a high replication rate may increase the frequency of mutations, which may lead to immune escape from vaccine-induced antibodies [10].

SARS-CoV-2 variants have rapidly emerged from the SARS-CoV-2 ancestral strain during the pandemic. These new variants cause surges in infection and a need for multiple booster doses to induce long-lasting protective immunity among immunocompetent as well as immunocompromised patients, including vulnerable dialysis patients.

The recent SARS-CoV-2 variants Delta and, later, Omicron show multiple mutations including the modulation of the ACE2-receptor-binding domain (RBD). These mutations cause reduced affinity which leads to immune escape and reduced vaccine efficacy [11,12]. Dialysis patients show weakened immune responses against the ancestral strain and also against variants, with a low or undetectable level of neutralizing antibodies against Delta and Omicron in 49% and 77% of these patients, respectively, after standard immunization [8,13].

In July 2021, ahead of other countries, the Israeli health authorities were quick to administer a third dose of the BNT162b2 vaccine (first booster dose). The administration of the booster dose was first approved for high-risk populations, including dialysis patients, and later for the whole population in an effort to halt the COVID-19 Delta variant wave [14,15,16]. Protection against COVID-19 spread was observed within 2 weeks following the booster, with a reduction in confirmed COVID-19 infection cases and reduced illness severity across all age groups [14,16,17].

In January 2022, during the B.1.1.529 (Omicron) variant wave and waning immunity [18], a second booster dose (fourth vaccine dose) was also approved by the Israeli authorities and later by the FDA and CDC. The immunogenic effect of the BNT162b2 vaccine second booster in dialysis patients is still unclear [19,20]. It is also unclear whether cross-reactivity against new variants can be induced by the BNT162b2 vaccine boosters. To this end, in the current study, we aimed to test the effect of the BNT162b2 booster doses on both humoral and cellular immunity and on SARS-CoV-2 infection rate among dialysis patients compared with age-matched volunteers without kidney disease.

We show that the first booster dose dramatically improved the levels of spike S1-RBD specific antibody titer, variant-specific neutralizing antibodies and T-cell responses in dialysis patients and volunteers. Furthermore, patients who were vaccinated with a second booster presented a tendency towards a reduced infection rate.

## 2. Materials and Methods

This is a single-center prospective comparative study in continuation with our previous studies evaluating the effectiveness of BNT162b2 in hemodialysis patients [19,20]. Overall, we recruited 100 patients on maintenance hemodialysis treatment from the Nephrology Department at Rabin Medical Center who had received two doses of the BNT162b2 vaccine and a booster dose from March 2021 onwards, and these patients were followed in our Nephrology Department until March 2022 [3,4].

The study was approved by the Rabin Medical Center (RMC) Institutional Ethics Committee (#RMC 0036-21). Informed consent was obtained from all subjects involved in the study.

The 100 hemodialysis patients who were included in the current final cohort were compared with 66 age-matched control non-dialysis volunteers with a normal range of the estimated glomerular filtration rate (eGFR). All of the participants received a third BNT162b2 vaccine dose according to the Israeli Ministry of Health recommendations, at least 5 months after the second dose.

In January 2022, high-risk populations (including this study’s patients) were encouraged by the Israeli Health Organization to receive a fourth vaccine dose (a second booster), around 150–160 days on average after the first booster for the Israeli population. We continued collecting data on vaccination preferences and the infection rate of patients and volunteers that were vaccinated or not with the fourth dose.

The infection rate was monitored and the breakthrough of SARS-CoV-2 infection events was diagnosed by PCR test. Data were collected from the electronic medical records of all participants.

### 2.1. S1-RBD Antibody Titer

Plasma samples for anti-spike1 antibodies were collected before and 2–3 weeks after the first booster dose.

Blood samples for anti-spike antibody levels, neutralizing antibodies and PBMCs were collected from the dialysis patients before a routine hemodialysis treatment, and from the control volunteers in our nephrology clinic.

All samples were immediately centrifuged at 3000 RPM and stored at −20 °C. The SARS-CoV-2 IgG II Quant (Abbott©) assay was used for quantitative measurement of IgG antibodies against the receptor-binding domain (RBD) of the spike1 protein (Anti-S1-RBD IgG). The test was considered positive if IgG was 50 AU/mL and above, in accordance with the manufacturer’s instructions.

Clinical, demographic and laboratory data (biochemical profile) and vaccine adverse events were obtained by means of questioning and from electronic medical records. SARS-CoV-2 infection was confirmed by polymerase chain reaction (PCR). The day of infection was the date of the first positive PCR test result. All of the PCR tests were performed according to clinical indications.

### 2.2. Stimulation of CD4+ and CD8+ T Cells

The cellular immune response was analyzed using flow cytometry analysis (FACS) of the wild-type (WT) spike protein as well as the Delta variant spike protein and N protein in a sub-cohort of this study population (n = 36, 23 dialysis patients and 13 controls) 4 months after the first booster vaccination (Table S1).

We freshly isolated peripheral blood mononuclear cells (PBMCs) by Ficoll gradient centrifugation. We used SARS-CoV-2 T Cell Analysis Kit (PBMC) human (#130-128-034) (Miltenyi Biotec, Ergisch Gladbach, Germany).

PBMCs (10^6^ per mL) were stimulated with SARS-CoV-2 peptide pools: S pool (PepTivator SARS-CoV-2 Prot_S 130-126-700#), S1 wild type (PepTivator SARS-CoV-2 Prot_S1, Delta spike (PepTivator, SARS-CoV-2 Prot_S) B.1.617.2) and nucleoprotein antigen (PepTivator SARS-CoV-2 Prot_N 130-126-698#).

PBMC samples of healthy donors and dialysis patients were incubated for 4 h with the indicated different SARS-CoV-2 PepTivator peptide pools in the presence of brefeldin A according to the manufacturer’s protocol. As negative controls, samples were left untreated with water/DMSO. Subsequently, T-cell lineage surface markers and intracellular cytokines were stained with eight fluorochrome-conjugated antibodies according to the manufacturer’s protocol: anti-CD3, anti-CD4, anti-CD8, anti-CD154 (CD40L), anti-IFN-γ, TNF-α, anti-IL2 anti-CD14, anti-CD20 and a live dead marker (Viobility 405/452 Fixable Dye). Flow cytometry was performed using a Beckman Coulter flow cytometer. Analysis was performed using Kaluza analysis software. Responses were measured against the wild type: SARS-CoV-2 WA/2019 or B.1.617.2 (Delta) variants. The results in PBMCs from unvaccinated, uninfected individuals or after infection were also tested as controls. Media backgrounds were subtracted from the specific values. We included only cases with a negative response to the N antigen.

### 2.3. Neutralizing Antibodies against the SARS-CoV-2 Delta and Omicron Variants Following BNT162b2 Booster Vaccination

We studied neutralizing antibodies against COVID-19 variants: B.1.1.529 (Omicron) and B.1.617.2 (Delta) by means of the competition ELISA assay. The assay detects percentage inhibition of ACE2 activity by neutralizing antibodies to SARS-CoV-2 in the patient sera. Serum samples were collected at baseline and 2–3 weeks following the booster and were kept frozen after studying anti-S antibodies. According to the manufacturer’s recommendations, the samples were heat-treated for 30 min at 56 °C, diluted to 1:10 and incubated with B.1.617.2 SARS-CoV-2 HRP spike (#RAS-N040-96 ACRO Biosystems, Newark, DE, USA) or B.1.1.529 spike (#RAS-N056 ACRO Biosystems). The presence of neutralizing antibodies was tested in samples which compete with ACE2 for HRP-SARS-CoV-2 spike RBD binding. Neutralizing antibodies were calculated as the percentage inhibition of the positive and negative control according to the manufacturer’s protocol. A positive neutralizing antibody inhibition effect is considered to be above 20% binding.

### 2.4. Statistical Analysis

All results were analyzed by GraphPad Prism 8.4.3. Multiple group comparisons were analyzed by running parametric (ANOVA) statistical tests, Kaplan–Meier curve, *t*-test or Mann–Whitney test, as appropriate. We used the receiver operating characteristic (ROC) curve to compare different antibody assays.

## 3. Results

### 3.1. Details of the Study Population

Overall, 100 patients hemodialyzed at the dialysis units of the Rabin Medical Center were enrolled. Inclusion criteria included vaccination with a first booster dose of BNT162b2 (Pfizer–BioNTech), after the standard vaccination regimen (Table S1). As a control, we included 66 age-matched non-dialysis subjects with normal kidney function. The control group underwent the same standard vaccination regimen with a first booster dose.

The dialysis patients’ mean age was 72 ± 12 years; 70% were males, and 58% were diabetic (Table S1). The control non-dialysis group’s mean age was 71 ± 5 years, with 60% being males and only 10% being diabetic.

One hundred sixty days after the first booster, 31% of the hemodialysis patients were vaccinated with a second booster dose. Overall, 58% of the control volunteers received a second booster, according to the participants’ choice.

PCR tests indicated that 18% of the study population were infected with SARS-CoV-2 (29% of the dialysis and 17% of the controls). During the study, three mortality cases related to SARS-CoV-2 infection occurred in the dialysis group.

#### The Effect of Booster on the Infection Rate

**Figure 1:**
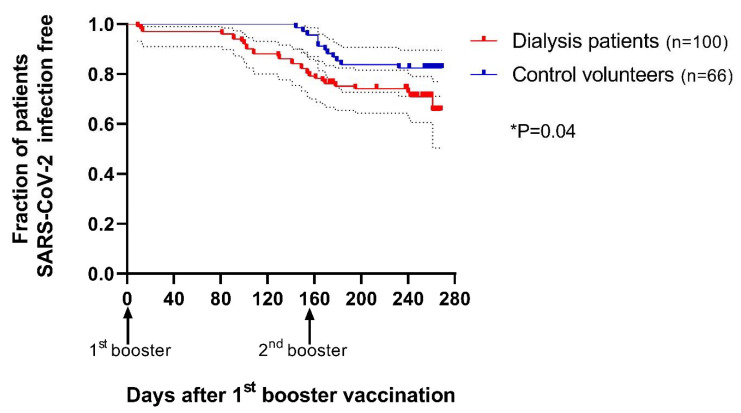
The infection rate was significantly higher in the dialysis patients after the booster dose.

The infection rate was significantly higher in the dialysis group, as demonstrated by Kaplan–Meier curve analysis (*p* = 0.04, Figure 1). In the time interval before the collection of serum for antibodies, two dialysis patients were confirmed as SARS-CoV-2-positive.

Fraction of 100 dialysis patients (red) and 66 control volunteers (blue) with SARS-CoV-2 infection after the first booster vaccination (95% CI).

**Figure 2:**
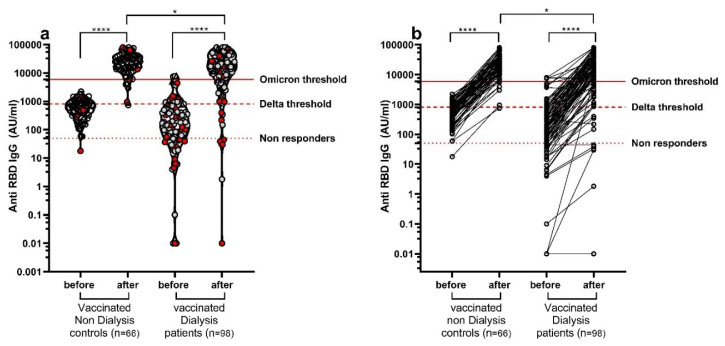
Anti-SARS-CoV-2 spike1 IgG titer was significantly upregulated after the first booster. Anti-SARS-CoV-2 spike protein IgG antibody titer in hemodialysis patients and controls before and 2–3 weeks after the first booster. (**a**) In red: patients and volunteers infected with SARS-CoV-2 after the booster dose during the 280-day study follow-up. (**b**) Changes in antibody titers in each participant. All dialysis patients but one showed upregulation of anti-S1 antibodies. * *p*  <  0.05; **** *p*  <  0.0001.

The anti-SARS-CoV-2 spike1 IgG antibody titer was significantly upregulated after the booster. Only five dialysis patients remained seronegative (under 50 AU/mL) following the first booster. The S1-RBD-IgG titer increased significantly following the booster from 686 AU/mL (CI 421–686) to 28,909 AU/mL (CI 16,599–28,909) in the control and from 285 (CI 110–285) AU/mL to 21,244 (CI 11,287–21,244) AU/mL in the dialysis group. Following the booster, 26% of the dialysis patients remained under the Omicron threshold of 5889 AU/mL (calculated anti-S1 titer that represents 20% neutralizing antibodies) and 8% remained under the Delta threshold of 809 AU/mL. In the control group, only 6% remained under the Omicron threshold and none remained under the Delta threshold (Figure 2).

We estimated the infection risk based on the anti-S1 titer before infection [21]. We found that 37.5% of the dialysis patients that were infected after the booster showed an antibody titer under the Omicron threshold compared to only 13% of the uninfected dialysis patients (Figure 2a).

### 3.2. Cellular Immune Response after Vaccine Booster Is Decreased in Hemodialysis with Preserved Effect against Delta Variant

**Figure 3:**
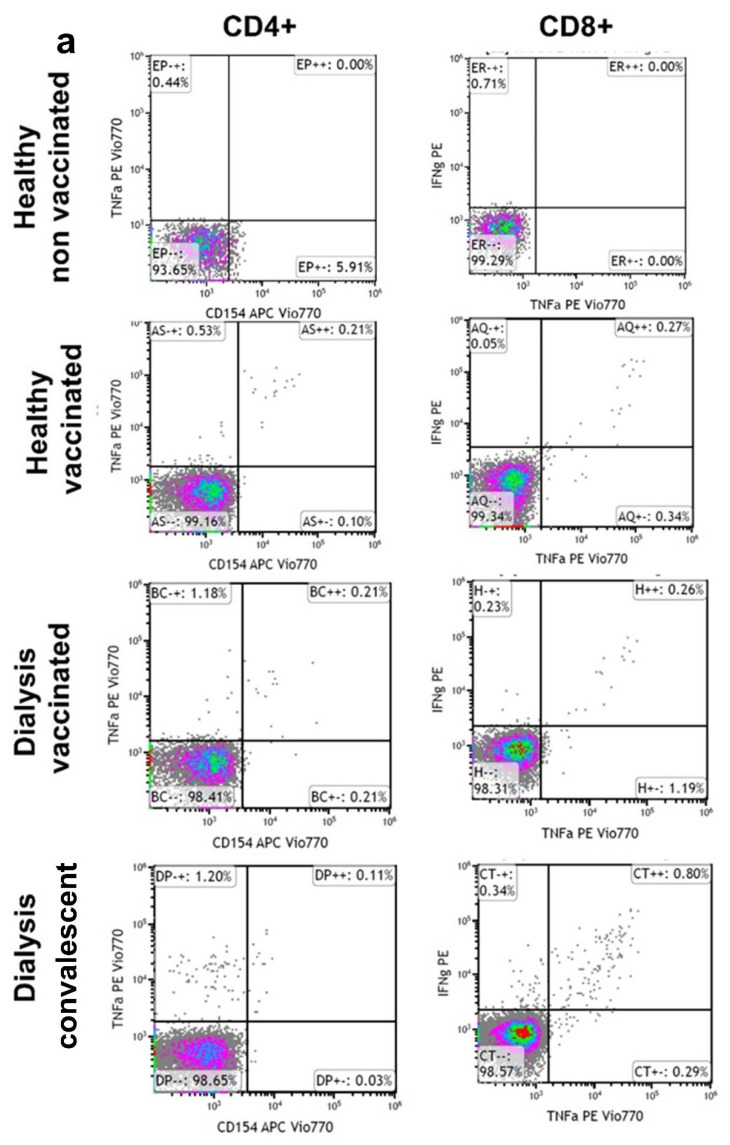

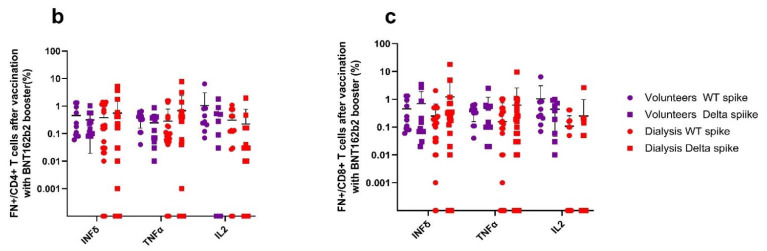
Cellular immune response to wild-type (WT) spike and Delta spike. Spike peptide-specific responses by intracellular cytokine staining assays in CD8+ T cells and CD4+ T cells (**a**–**c**) 4 months after vaccination in dialysis patients (●■) or volunteers (●■). Means of CD4 or CD8 % population presenting a response (**b**,**c**) are numerically shown. CD4+ (**b**) CD8+ T cells’ (**c**) production of IL2, TNFα and IFNγ against WT spike ●● and B.1.617.2 (Delta) ■■. Media backgrounds were subtracted from the specific values. Representative dot blot FACS analysis (**a**). CD4+ or CD8+ T cells’ response to spike stimulation in PBMCs from dialysis patients compared to the healthy cohort. Additionally, shown are cellular response data from unvaccinated, uninfected individuals, or after infection (convalescent).

We next examined whether vaccinated hemodialysis patients show cellular immunity similar to that of non-dialysis volunteers 4 months after the first booster dose (Figure 3a). We additionally examined whether mutations disrupted the total level of CD4+ and CD8+ T-cell response. We analyzed the spike-reactive T-cell response in PBMCs from dialysis patients and the control age-matched cohort. Dialysis patients demonstrated lower frequencies of WT and Delta spike-reactive CD4+ and CD8+ T cells compared with the control volunteers. Only 70% of the dialysis patients showed any CD8 cell interferon-γ secretion compared with 100% in the vaccinated volunteers. The production of IL2, TNFα and IFNγ by SARS-CoV-2-reactive CD4+ T cells presented a similar pattern, with a higher response of CD8+ cells.

The T-cell response to the Delta variant was preserved in those dialysis patients and controls that were reactive to the WT, with no significant decrease in cytokine response for the Delta variant.

### 3.3. Neutralizing Antibodies against COVID-19 Variants in Hemodialysis Patients and Volunteers

To further assess vaccine effectiveness, we studied neutralization antibodies against COVID-19 Delta (B.1.617.2) and Omicron (B.1.1.529) variants.

Neutralizing antibodies were evaluated in the sera of 34 dialysis patients (34% of all dialysis study population) (Table S1) in the same samples used for anti-S1 antibodies.

**Figure 4:**
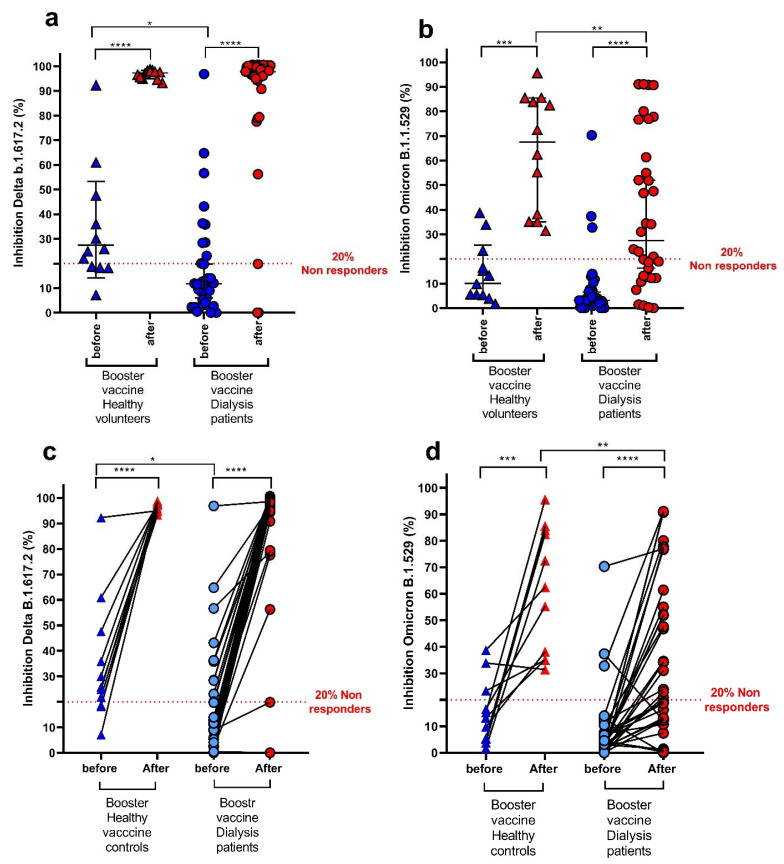

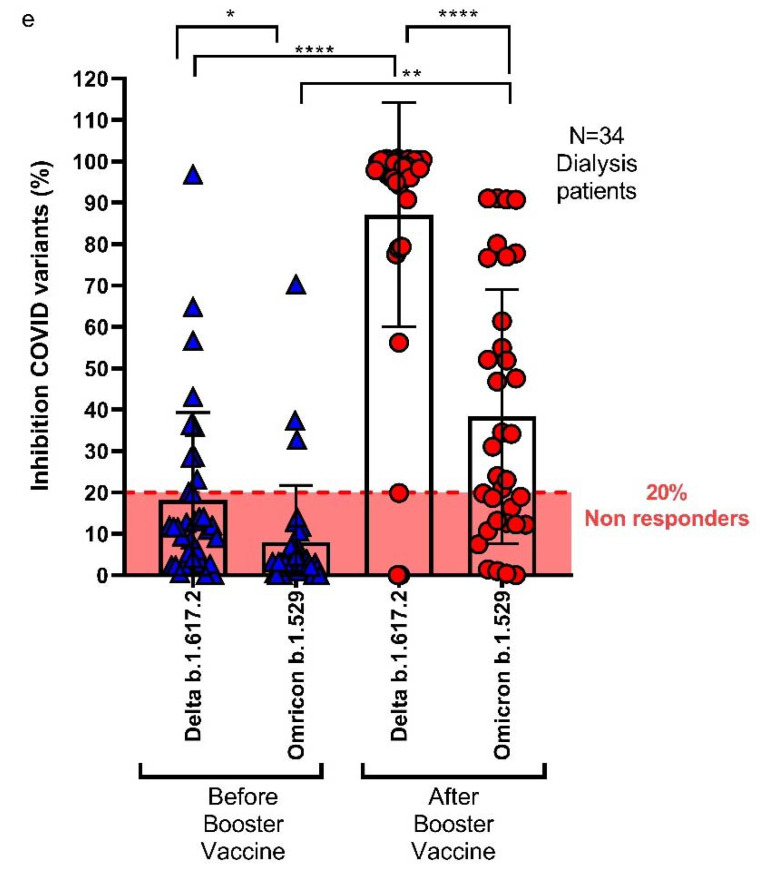
(**a**–**d**) Plasma neutralization titers against Delta (**a**,**c**) and Omicron (**b**,**d**) in dialysis patients (n = 34) and controls (n = 12) who received booster doses of the mRNA vaccine showing results for plasma neutralization titers measured before and 2–3 weeks after the first booster dose. (**e**) Neutralizing antibodies in dialysis patients only against Delta or Omicron. The graph represents mean  ±  SD. * *p* <  0.05; ** *p* <  0.01; *** *p* <  0.001; **** *p* <  0.0001.

Neutralizing antibody inhibition against the Delta variant at baseline was 33.5 ± 23.5% in the control group and 18.9 ± 22.6% for the dialysis group (*p* = 0.064), and it was increased after the booster dose to 96.7 ± 1.8% and 86.4 ± 27.6%, respectively (Figure 4a,c).

Before receiving the first booster, 10 out of 34 dialysis patients (29.4%) and 8 out of 12 (66.7%) controls had neutralizing antibody activity against the Delta variant (*p* = 0.023). After the booster, 91.2% (31 patients) of the dialysis group and 100% of the control group were above the threshold of 20% inhibition (Figure 4a,c).

The neutralizing activity against the Omicron variant at baseline was 8.0 ± 13.7% in the dialysis group and 14.4 ± 12.0% in the control group (*p* = 0.163) and increased only to 38.3 ± 30.7% and 63.6 ± 23.8%, respectively, with a significantly lower response in the dialysis patients compared to the control group (*p* = 0.008) (Figure 4b,d).

The BNT162b2 booster resulted in a 4.5-fold increase in neutralization activity against Delta and a 4.8-fold increase against Omicron (Figure 4). However, despite this increase, neutralizing antibody titers were inferior by 2-fold for Omicron compared to the Delta variant (Figure 4e).

#### 3.3.1. Comparison of Antibody Titer and Neutralizing Activity by SARS-CoV-2 Breakthrough Infections after the Booster Dose

We next categorized the dialysis patients compared to controls by their SARS-CoV-2 infection status (if they were infected after the booster) and assessed whether neutralizing antibodies against Omicron predicted infection risk. Nine out of forty-six patients and controls were infected with Omicron after the booster. There was no difference between the neutralizing antibody titer against Omicron and the risk of infection breakthrough (9% and 44% in infected patients vs. 10% before and 49% after booster in non-infected patients, respectively).

#### 3.3.2. Comparing Anti-S Antibodies and Neutralizing Antibodies

**Figure 5:**
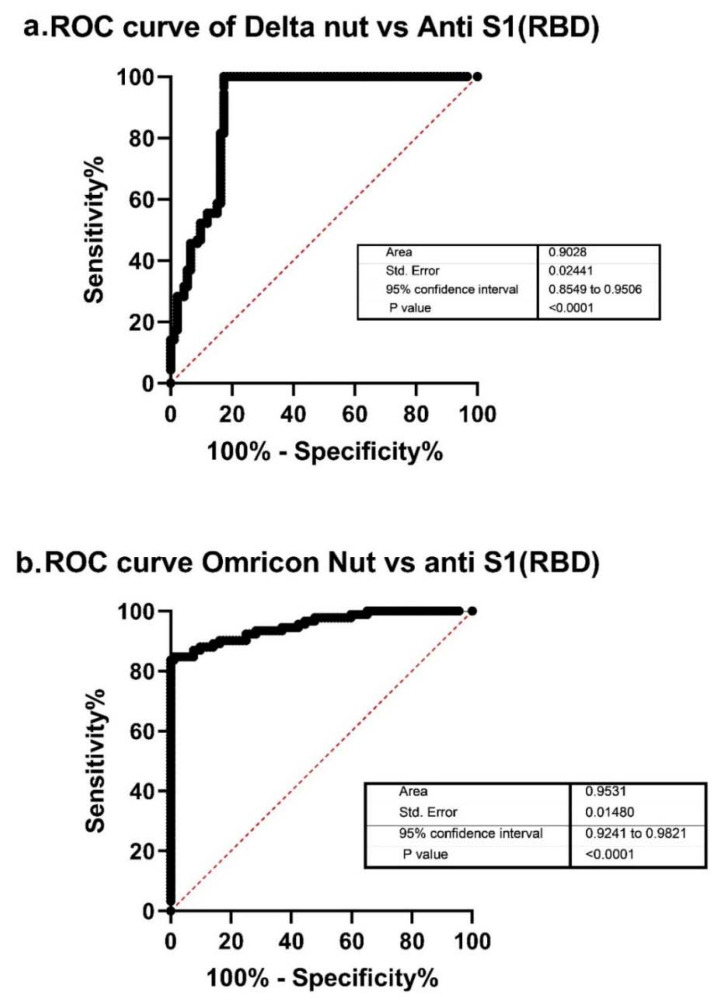
ROC of Delta neutralizing (nut) antibody and anti-S1 (RBD) antibody titers (**a**) and Omicron neutralizing (nut) antibody and anti-S1 (RBD) antibody titers (**b**).

The evaluation of anti-spike antibodies represents a useful tool to estimate individual protection. However, neutralizing antibodies are considered to be a more accurate test to assess immunity. The area under the receiver operating characteristic (ROC) curve was 0.9028 for Delta (95% CI, 0.8549–0.9506) and 0.9531 for Omicron (95% CI, 0.9241–0.9821) (Figure 5). Variant neutralization (20% of the virus-neutralizing antibody inhibition) values were 809 AU/mL for Delta and 5889 AU/mL for Omicron. These values in relation to anti-RBD IgG antibodies for the whole study population are presented in Figure 2.

### 3.4. The Effect of the Second Booster on Omicron Infection Rate

In January 2022, the Israeli authorities approved the administration of a fourth vaccine dose: a second BNT162b2 vaccine booster dose (140–160 days after the first booster for this study population). Overall, 58% of the volunteer control group were vaccinated with the second booster dose, compared with only 47% of the dialysis patients.

The dialysis patients with the second booster showed a trend of reduced infection rate that was not statistically significant by the end of this study. The second booster dose significantly reduced the infection rate in the control and dialysis groups pooled together (*p* = 0.02).

**Figure 6:**
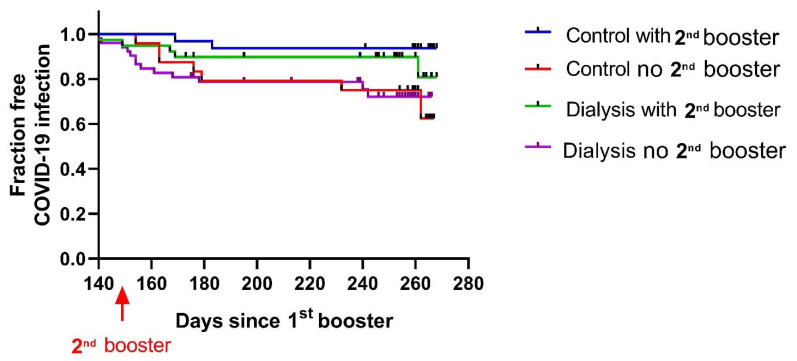
Fraction of SARS-CoV-2 infection after the second booster vaccination in dialysis patients (number at risk N = 90) and volunteers (number at risk N = 56) that were not infected until the time of starting second booster administration. Volunteers with (blue line) or without (red line) a second booster vaccination; dialysis patients with (green line) or without (purple line) a second booster.

In the healthy cohort, 6% of the subjects who were vaccinated with the second booster were infected, compared with 22% among those who were not vaccinated with the second booster. In the dialysis group, 13% versus 25% were infected, respectively (Figure 6).

## 4. Discussion

BNT162b2 vaccine-induced antibodies decline rapidly in the general population as well as in dialysis patients [4,6]. The emergence of new COVID-19 variants along with the waning of the protective antibody titer [3,4,18] raised the need for a booster dose of the vaccine [20,22].

In this study, we aimed to evaluate the effectiveness of a BNT162b2 booster vaccine against the new COVID-19 variants in dialysis patients compared to non-dialysis age-matched controls.

We report here that the booster dose, given after the standard vaccination regimen, dramatically improved the levels of wild-type S1 (RBD) specific antibodies and significantly improved the levels of Delta- and Omicron-specific neutralizing antibodies. Furthermore, a second booster may reduce the outbreak of infections with further coronavirus variants.

Dialysis patients have been previously shown to have a decreased immune response to any vaccine, including the BNT162b2 vaccine [4,23]. In this study, we show that despite the increase in the anti-SARS-CoV-2 spike IgG antibody titer (Figure 2) [2,3,4,24] and in the neutralizing antibody titer after vaccination in the dialysis patients, the vaccine effectiveness was inferior in these patients compared with non-dialysis controls (Figure 4).

The rate of infection was significantly higher among the dialysis patients compared to controls, measured at 300 days after the booster dose (Figure 1). Furthermore, more dialysis patients showed anti-S1 antibodies and neutralizing antibodies which were below the protective threshold when compared to healthy controls. Dialysis patients that were infected after the booster tended to have a lower anti-S1 antibody titer (Figure 2), pointing to the need for higher antibody titers to induce protection against Omicron and other new variants.

Since the Omicron variant contains up to 36 mutations, many of them in the spike receptor-binding domain (RBD), a decreased effectiveness of the vaccine which was designed against wild-type variant RBDs was expected [25]. Omicron-neutralizing antibodies (Figure 4e) did not predict Omicron infection risk, supporting the possibility that vaccine-induced antibodies may not be specific enough for the prevention of Omicron infection compared with wild-type or Delta variant infection [12,26].

Third doses of the BNT162b2 vaccine as a booster have become a worldwide policy. Studies have shown an overall improved humoral and cellular response in the general population as well as in immunosuppressed patients [14,16]. The booster enhances anti-wild-type spike antibodies in patients, with waning immunity after two doses. Our data also support the effectiveness of the booster dose in dialysis patients and elderly volunteers.

Booster doses of mRNA vaccine have been shown to reduce morbidity and mortality [14,16], increase humoral response and enhance neutralizing antibody response against the variants in healthy populations [26,27] as well as in dialysis patients [19]. In this study, we show an improved humoral response and cell immunity against the new variants in dialysis patients. Furthermore, we show a reduced infection rate at one-year follow-up in non-dialysis volunteers and a trend for reduced infection rate in the dialysis patients after a second booster.

Vaccination-induced neutralizing antibodies comprise a more sensitive and specific method for the evaluation of immunity against SARS-CoV-2 variants. We compared neutralization titers against Delta and Omicron variants in patients’ serum before and 2–3 weeks after the BNT162b2 booster. Although the booster was less effective against variants of SARS-CoV-2, it resulted in a 4-fold increase in neutralization activity against both Delta and Omicron. Neutralizing antibodies against Omicron were 2-fold lower compared with the Delta variant. Other reports have also shown reduced levels of neutralizing antibodies against SARS-CoV-2 variants [27]. We found no significant correlation between the levels of neutralizing antibodies and infection rate. Possible explanations are the small sample size and the prolonged time interval between antibody measurement and the infection episodes [28].

Complementary cellular (T cell) immune responses are activated following vaccination, mainly via CD8 T cells [28], which are considered more significant for long durable immune protection. Since T cells recognize a broader range of spike-protein-specific epitopes, cellular immunity can be more effective for the recognition of the spike protein of multiple SARS-CoV-2 variants [26]. We therefore studied spike-specific CD8+ and CD4+ T-cell responses in PBMCs of the dialysis patients. For this purpose, we analyzed differences between T-cell (CD4 and CD8) cytokine responses against wild-type spike variants or Delta spike variants.

We found that only 70% of the dialysis patients showed CD8+ cells with an increased capacity for cytokine secretion by spike activation in vitro compared to the entire control group (Figure 3a–c). However, we found no statistical difference between groups. In addition, we found a preserved response against the Delta variant compared with the wild-type virus (Figure 4). These results support the concept that the majority of CD4+ and CD8+ T-cell epitopes are unaffected by mutations, as seen in the Delta variant. We found a variable spike-specific T-cell response in dialysis patients that may be the consequence of chronic inflammation leading to T-cell overactivation or T-cell exhaustion. Our findings are in concordance with the data recently published by Shankar et al., who found no association between T-cell response and infection rate in dialysis patients [24].

Given the fact that the level of vaccine-induced T-cell response is very low (Figure 4), with high nonspecific inflammation and cytokine secretion which occurs in dialysis patients, it is possible that we have an over-diagnosis of cell immunity in dialysis patients.

In January 2022 (150–160 days after the first booster dose), all dialysis patients were offered by the Israeli health authorities a fourth vaccine dose as a second booster vaccination. Only 31% of the hemodialysis patients chose to be vaccinated with a second booster dose, compared to 58% of the healthy volunteers. Although our study groups are small, we found a decreased infection rate in those who received a second booster, similar to the data by Yinon et al. [14] that showed the effectiveness of the second booster in an Israeli population. The second booster was well tolerated with no major adverse events among all the populations.

The interpretation of our results on infection rate is limited by the small study size. In addition, infection diagnosis was based only on clinical data without routine PCR testing, with no data on anti-N antibodies, which may lead to under-diagnosis of asymptomatic infection cases.

Our study emphasizes the effectiveness of multiple booster vaccine doses during the waves of COVID-19 variant infections in dialysis patients. We demonstrated that booster doses in dialysis patients might also prevent new variant infections and improve sustained immune responses.

## 5. Conclusions

Repeated BNT162b2 boosters dramatically increase the intensity of humoral and cell-mediated immunity in dialysis patients, which tends to reduce the spread of new coronavirus variants.

## Data Availability

We declare data availability according to request.

## Supplementary Materials

The following are available online at https://www.mdpi.com/article/10.3390/vaccines10060967/s1, Table S1: Demographic data on the dialysis group in this study.
