# Supplementary material for: BNT162b2 Booster Vaccination Induced Immunity against SARS-CoV-2 Variants among Hemodialysis Patients
Source: Vaccines (Basel). 2022 Jun 17;10(6):967. doi: 10.3390/vaccines10060967 (PMC9227334; doi:10.3390/vaccines10060967)
Supplement: Supplementary file 1 [file vaccines-10-00967-s001.zip › vaccines-1740563-supplementary.pdf]

Table S1. Demographic data on the dialysis group in this study.

	Whole Dialysis group	Dialysis subgroup for neutralizing antibodiesand FACS	
Age(years)	71.8±12.2	71±12.8	NS
Gender(male%)	70%	65%	NS
Weight(Kg)	73.5±18	74±12	NS
Weight(cm)	166±25	167±8	NS
BMI(Kg/m2)	27.1±5.2	27.6±5.3	NS
T2DM(%)	58%	52%	NS
IHD(%)	50%	47%	NS
Malignancy(%)	22%	23.50%	NS
S/P transplant(%)	10%	8%	NS
Dialysis Efficiency (Kt/V)	1.4±0.3	1.5±0.3	NS
Protein catabolic rate (nPCR)	1.1±0.3	1.1±0.2	NS
Hb (g/L)	10.7±1.1	10.6±1	NS
Albumin(g/dL)	4±0.3	4±0.2	NS
Globulin(g/dL)	2.7±0.4	2.8±0.4	NS
